# Sexual Activities and Changes in Condom Use in Group Sex Events Among Female Sex Workers in Melbourne, Australia

**DOI:** 10.3389/fpubh.2022.834901

**Published:** 2022-04-06

**Authors:** Chen Lew, Christopher K. Fairley, Julien Tran, Tiffany R. Phillips, Ei T. Aung, Kate Maddaford, Marcus Y. Chen, Catriona S. Bradshaw, Eric P. F. Chow

**Affiliations:** ^1^Melbourne Sexual Health Centre, Alfred Health, Melbourne, VIC, Australia; ^2^Central Clinical School, Faculty of Medicine, Nursing and Health Sciences, Monash University, Melbourne, VIC, Australia; ^3^Centre for Epidemiology and Biostatistics, Melbourne School of Population and Global Health, The University of Melbourne, Melbourne, VIC, Australia

**Keywords:** sexual practice, sexual behavior, sex workers, commercial sex, group sex, threesomes, sexually transmitted infection, sexually transmitted disease

## Abstract

**Background:**

There are few studies investigating group sex among female sex workers (FSWs). This study aimed to examine the typical number of group sex participants, sexual activities and condom use in group sex among FSWs attending a sexual health clinic in Melbourne, Australia.

**Methods:**

We conducted a cross-sectional survey between May 1, 2019 and March 13, 2020 among FSWs in Melbourne. Participants were asked whether they had participated in group sex (sex involving more than two participants) within the past 3 months, the size of the most recent group sex event, sexual activities they engaged in and condom use. It is unknown whether this was a paid or unpaid event in our study.

**Results:**

Of the 247 FSWs surveyed, the mean age was 28.9 years and 51.8% were born in Australia. More than a quarter (27.5%, *n* = 68) had had group sex in the past 3 months with the number of events ranging from 1 to 10 times. The median size of the group was 3 participants with one man and two women (including the FSW) being the most common combination. Kissing was the most common activity in group sex. Of 58 FSWs who had vaginal sex during group sex, 77.3% (51/58) reported their male partners always used condoms but 13.7% (7/51) of these did not change condoms between women.

**Conclusion:**

This study finds that group sex is common among FSWs. Although condoms are always used during group sex, one in six men did not change their condoms between partners, which may increase STI transmission between partners.

## Introduction

Group sex, a sex event that involves more than two participants, has been considered a high-risk activity for the acquisition of HIV and other sexually transmitted infections (STIs) due to a greater number of partners affected by condomless sex (1). Previous studies have estimated about 27.2–34.8% of gay, bisexual and other men who have sex with men (MSM) had participated in at least one group sex event in the past 3 months (2, 3). An exploratory study by Turek et al. (4) reported 49% of 51 female sex workers (FSWs) had participated in at least one group sex event in the past 3 months. However, given their small sample size, results from this study may have been limited.

There are few studies investigating group sex among FSWs. While most group sex studies have focused on MSM, other at-risk populations such as heterosexuals and FSWs have been understudied (2, 3). Additionally, previous studies have predominantly examined the association between group sex participation and HIV/STI acquisition with few data on the details of sexual activities during the group sex event such as the size of the group sex event (i.e., the number of participants) and the sexual activities involved during group sex. Furthermore, the majority of studies collected and reported condom use during group sex as a binary variable (yes or no) (2, 4, 5) and it is unclear whether the participants used a new condom when they changed partners. Using the same condom for more than one partner during group sex increases the risk of STI and HIV transmission between partners (6). To our knowledge, only one study has investigated whether MSM changed condoms in between switching partners during group sex, which was undertaken in the United States (7), while none have explored this practice among FSWs. As previous studies have estimated that almost half of FSWs participate in group sex but no previous studies have extensively investigated the sexual activities and practices during these sex events among FSWs, further exploration into the risks of group sex participation in this population is required to further guide safe sex practices (2, 4, 5). Therefore, we aimed to explore characteristics of group sex among FSWs, including the number of people involved, sexual activities and condom use in between switching partners.

## Methods

This was a cross-sectional study conducted at the Melbourne Sexual Health Center (MSHC). MSHC is a public sexual health clinic based in Melbourne, Australia. All clients attending the MSHC for the first time or have not been seen for 3 months are asked to complete a routine questionnaire using computer-assisted self-interviewing (CASI). This questionnaire collects information on demographic characteristics and sexual practices for routine clinical care and management. After completing CASI, eligible clients were invited to participate in a voluntary survey called the “Kissing And Sexual Practice (KASP)” survey between May 1st, 2019 and March 13th, 2020. Our study originally aimed to recruit at least 200 FSWs who had participated in group sex; however, the study was ceased earlier due to the COVID-19 pandemic as sex workers were not permitted to work during the repeated COVID-19 lockdowns in Melbourne. This study was approved by the Alfred Hospital Ethics Committee, Melbourne, Australia (647/17).

To be eligible to participate in the KASP survey, individuals must be female, aged 18 years or above, read English (while the CASI was available in different languages, the KASP survey was only available in English) and self-reported currently working as a sex worker at the time they were invited to participate in the survey. Transgender individuals were also excluded from the final analysis. Sex work law varies between states in Australia (8). In Victoria, sex work has been legalized under the Sex Work Act since 1994 (9). Regulations require sex workers to use condoms with any paid sexual activity, and undergo a 3-monthly screening for HIV, syphilis, chlamydia, and gonorrhea (9, 10).

Consent was obtained from participants by selecting “yes” indicating they were agree to participate before the commencement of the KASP survey. The KASP survey included questions on the number of group sex events in the past 3 months, the number of men and women involved in the most recent group sex event, the number of different sexual activities they engaged in (i.e., tongue kissing, oral sex, vaginal sex, and anal sex), condom use during sex, and whether men changed condoms between partners (“never,” “sometimes,” “always,” or “did not know”). We defined group sex as participation in at least one sexual activity with more than two persons excluding the study participant in the past 3 months. We did not specify whether the group sex event was with paying clients or non-paying private partners. All FSWs attending the MSHC were offered HIV and STI (including gonorrhea, chlamydia, and syphilis) screening. Data on HIV and STI diagnoses on the day they completed the survey were also extracted from the clinic electronic database.

Continuous variables were summarized using mean, standard deviation, median and interquartile range. Categorical variables were summarized using frequency and proportion. We compared the characteristics between FSW who had had group sex and FSW who did not have group sex using a two-sided *t*-test for continuous variables and Fisher's exact test for categorical variables. We performed three regression models to examine whether the demographic characteristics (age and country of birth) were associated with (1) the number of group sex episodes; (2) number of sexual partners in the most recent group sex; and (3) the change of condoms between partners in the most recent group sex. Negative binomial regression model was used to identify any explanatory characteristics related to the number of group sex episodes, and the number of sexual partners; while logistic regression model was used to any explanatory characteristics related to the change of condoms between partners in the most recent group sex. All statistical analyses were performed using Stata v17 (StataCorp. 2021. Stata Statistical Software: Release 17. College Station, TX: StataCorp LLC.).

## Results

Between May 2019 and March 2020, 1,104 elegible individual FSWs were invited to participate in the KASP survey, 247 (22.4%) FSWs consented to participate and were included in the final analysis. There were no significant differences in mean age, country of birth and proportion of injecting drug use in the past 3 months between those who participated in KASP and those who did not.

Of the 247 FSWs, the mean age was 28.9 years (SD: 7.7) and more than half (51.8%, *n* = 128) were born in Australia. More than a quarter (27.5%, *n* = 68) had group sex in the past 3 months and the number of group sex events ranged from one to ten, with a median of two (IQR: 1–3).

There was no significant difference in age, injecting drug use, and HIV and STI test positivity between those who had and did not have group sex in the past 3 months (Table 1). Furthermore, there was no significant difference in any STI positivity (i.e., gonorrhea, chlamydia, or syphilis) or each specific STI positivity between those who changed condoms and those who did not change condoms between partners during group sex. However, those who were born in Australia were significantly more likely to participate in group sex (*p* = 0.004). Age and country of birth were not associated with the number of group sex episodes in the past 3 months in the negative binomial regression.

**Table 1:** Demographic characteristics, HIV and STI test positivity between female sex workers who had group sex compared to those who did not.

	**Had group sex**	**No group sex**	***P-*value**
	**(*N* = 68)**	**(*N* = 179)**	
**Demographic characteristics**			
Age, mean (SD)	29.3 (7.5)	28.8 (7.8)	0.681
Country of birth, *n* (%)			0.004
Australia	45 (66.2%)	83 (46.4%)	
Overseas	18 (26.5%)	80 (44.7%)	
Unknown	5 (7.4%)	16 (8.9%)	
Injecting drug use in the past 3 months, *n* (%)			0.574
Yes	1 (1.5%)	4 (2.2%)	
No	67 (95.5%)	173 (96.6%)	
Unknown	0	2 (1.1%)	
**HIV/STI test positivity**, ***n*** (%)*			
Gonorrhea	2/64 (3.1%)	2/176 (1.1%)	0.607
Chlamydia	1/63 (1.6%)	2/176 (1.1%)	0.602
Syphilis	0/56	3/149 (2.0%)	0.382
HIV	0/56	0/148	
Any HIV/STI	3/64 (4.7%)	7/176 (4.0%)	0.526

*SD, standard deviation; HIV, human immunodeficiency virus; STI, sexually transmitted infections.*

*Not all individuals were tested for HIV or STIs, the data are presented as n/N (%), where n is the number of women tested positive, N is the number of women tested and % is the test positivity. Positive STI result indicates they were positive on the day they completed the KASP survey. Any HIV/STI positivity was defined as the individual was tested positive for HIV, syphilis, gonorrhea or chlamydia.

Of the 68 FSWs who had had group sex, the total number of people involved in their most recent group sex event ranged from three to eight participants with a median of three, with the most common combination being one man plus two women including the FSW (Table 2). There was one FSW who had had group sex that involved two other women without any men. Participant's age and country of birth were not associated with the number of sexual partners in the most recent group sex.

**Table 2:** Total number of individuals participating in the most recent group sex event among 68 female sex workers.

	**Range**	**Median (IQR)**	**Mean (SD)**
Total number of individuals (including the FSW)	3–8	3 (3–5)	4.2 (1.7)
Total number of men	0–4	1 (1–2)	1.4 (1.0)
Total number of women (including the FSW)	1–5	2 (2–3)	2.5 (1.0)

*IQR, interquartile range; SD, standard deviation*.

During the most recent group sex activity, almost all FSWs (97.1%, *n* = 66) had kissed, with a median number of kissing partners of 2 (IQR 2–4) (Table 3). The median number of male kissing partners was the same as the median number of female kissing partners. The second most common activities were oral sex and vaginal sex. There were 58 FSWs (86.6%) who performed fellatio on men. There were 58 FSWs (86.6%) that received cunnilingus from women and 54 FSWs (80.6%) that received cunnilingus from men. There were 58 FSWs (86.6%) who had vaginal sex and eight FSWs (11.8%) who had anal sex with men in their most recent group sex event.

**Table 3:** Different types of sexual activities involved in the most recent group sex among 68 female sex workers.

**Sexual activities**	**Number and proportion of FSWs who engaged in each activity**		**Number of partners engaged in each activity**	
	***n***	**%**	**Range**	**Median (IQR)**	**Mean (SD)**
Kissing anyone	66	97.1%	0–7	2 (2–3)	2.6 (1.5)
Kissing with men	53	77.9%	0–4	1 (1–2)	1.2 (0.9)
Kissing with women	62	92.5%	0–5	1 (1–2)	1.6 (1.1)
Performing fellatio on men	58	86.6%	0–5	1 (1–2)	1.5 (1.1)
Receiving cunnilingus from women	58	86.6%	0–5	1 (1–2)	1.3 (1.0)
Receiving cunnilingus from men	54	80.6%	0–5	1 (1–2)	1.3 (1.0)
Vaginal sex with men	58	86.6%	0–5	1 (1–2)	1.6 (1.1)
Anal sex with men	8	11.8%	0–3	0 (0–0)	0.18 (0.55)

*IQR, interquartile range; SD, standard deviation*.

**Figure 1:**
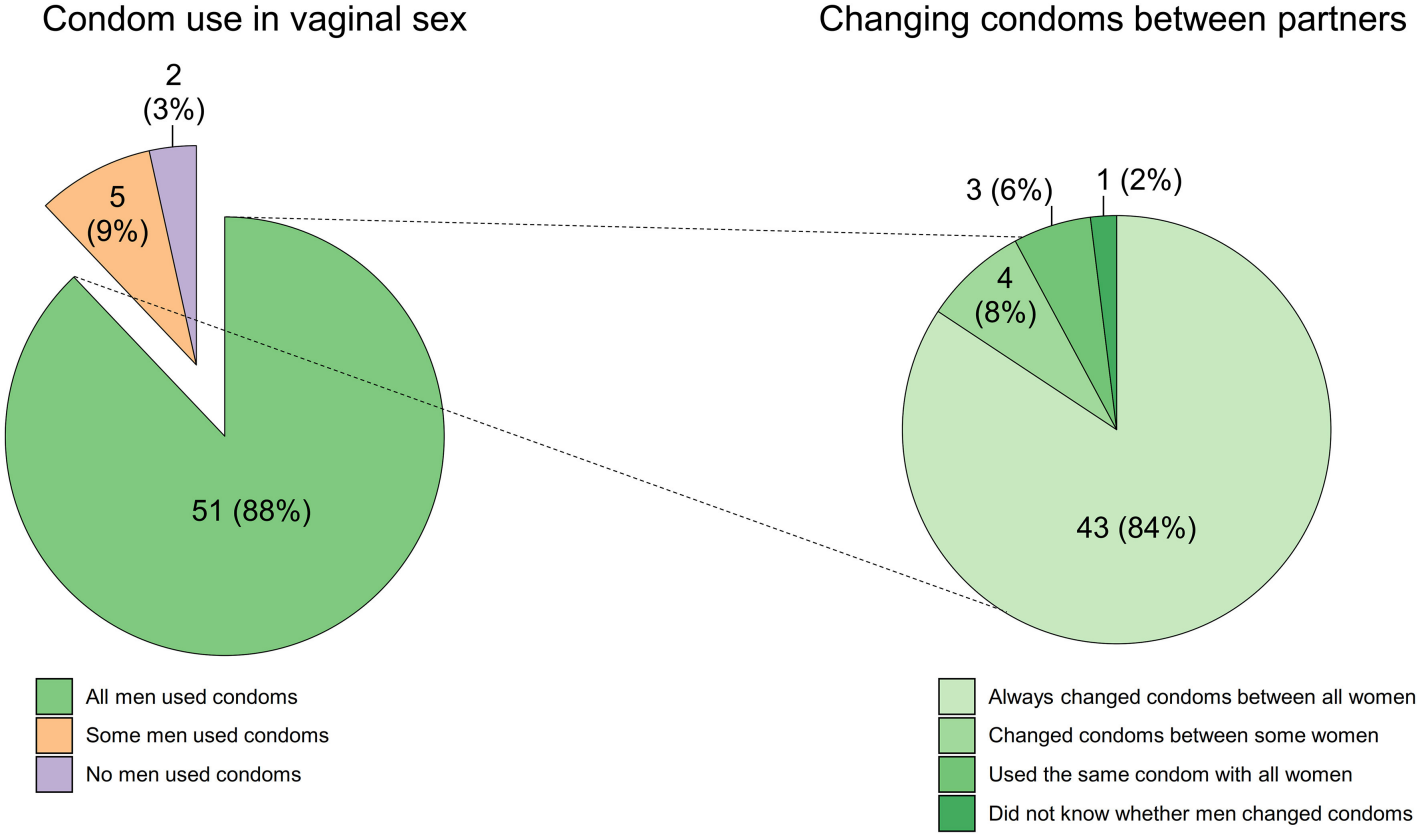
Condom use among 58 female sex workers who had vaginal sex in their most recent group sex event, with a subset detailing condom practice among the 51 female sex workers who used condoms with all men.

Of the 58 FSWs who had vaginal sex, 51 (87.9%) reported always using condoms, five (8.6%) reported sometimes using condoms and two (3.4%) reported never using condoms. Of the 51 FSWs who reported always using condoms with all men, 43 (84.3%) reported their male partners changed condoms between all women, four (7.8%) reported their male partners only changed condoms with some women, and three (5.6%) reported their male partners used the same condoms with all women (Figure 1). Furthermore, of those who reported always using condoms during vaginal sex, 33 (64.7%) performed fellatio with their male partner after having vaginal sex. Of the seven FSWs who had condomless vaginal sex with men (including participants who changed condoms between some men only), all (100%) performed fellatio after condomless vaginal sex. It is unknown whether fellatio was performed with condoms or not, as this was not a question in our survey.

Of the eight FSWs who had anal sex, five (62.5%) reported always using condoms and three (37.5%) reported never using condoms. Of the five FSWs who reported always using condoms, four reported their male partners changed condoms between all women, and one reported her male partners only changed condoms with some women. Furthermore, of those who reported always using during anal sex, 3 (60.0%) performed fellatio with their male partner after having anal sex. Of the three FSWs who had condomless anal sex with men, two (66.6%) performed fellatio after condomless anal sex.

Logistic regression analyses showed that the demographic characteristics (i.e., age and country of birth) were not associated with the change in condom use in both vaginal and anal sex in the most recent group sex.

## Discussion

In our study of 247 FSWs attending a sexual health clinic in Melbourne, Australia, we found that more than a quarter (27.5%) had engaged in group sex in the past 3 months, most commonly with a total of three participants, with one man and one woman with the FSW being the most common combination. Kissing was the most common activity in group sex, and most had fellatio, cunnilingus and vaginal sex. One in ten FSWs reported anal sex in their most recent group sex. Consistent condom use for vaginal sex in the most recent group sex activity was high (77%) but one in six men did not change condoms between each woman which can increase STI transmission between partners. There was a high proportion of FSWs who performed fellatio after condomless vaginal or anal sex whereby STIs may be transmitted across anatomical sites.

We found that more than a quarter of FSWs in our study had engaged in group sex in the past 3 months, which is lower than a previous study conducted at the same clinic by Turek et al. (4). In Turek et al.'s study, they surveyed 51 FSWs in March and April 2019, and found that 49% of FSWs reported having group sex in the past 3 months (4). The higher proportion reported in Turek's study may be attributed to its small sample size due to low variation in point estimates. Similarly, Turek's study did not investigate whether their last group sex event occurred at work, and further studies may be warranted to investigate this relationship further. Consistent with Turek's study, demographic characteristics of age, gender and injecting drug use were not associated with group sex (4). However, Australian-born FSWs were significantly more likely to engage in group sex compared to overseas-born FSWs. Our clinic commonly sees sex workers from more conservative cultural backgrounds (such as Chinese and Thai), and a theory to this increased group sex participation amongst Australian-born FSWs may be that different cultural attitudes toward same-sex practice may make some FSWs less likely to participate in group sex. Therefore, further studies may be warranted to investigate group sex amongst FSWs from different cultural groups to establish and explore this association further.

Most FSWs (77%) reported that their male partners always used condoms for vaginal and anal sex in the most recent group sex event. While this is proportion is lower than reported in the literature, where evidence suggests that condom use amongst FSWs during work is >90%, we could not distinguish whether the group sex was paid work from this survey, and group sex amongst private partners may account for this difference (11). However, while condom use amongst FSWs is high, one in six men did not change condoms for vaginal sex between women and one in five men did not change condoms for anal sex between women. Using the same condom is used between women increases the risk of STI transmission (6). Phillips et al. surveyed 503 MSM in 2014 in the United States, and found that one-quarter of MSM did not change condoms between partners during group sex, and this was associated with an increased risk of STI transmission (2). Additionally, while not collected in our study, it may also be important to use a new condom when changing between oral, vaginal and anal sex with the same partner as STIs can be transmitted between anatomical sites (12). A Peruvian study on 120 FSWs in 2014 has found that only 50% of FSWs changed condoms between vaginal and anal sex with clients, and this dropped to 15% with private partners (13). Studies have shown that cross-contamination from different mucosal sites increases STI transmission risk to themselves from other sites of the body, and that vaginal sex following anal sex also increases the risk of feculent matter entering the genital tract, which increases the risk of conditions such as bacterial vaginosis due to contaminant bacteria (14). There has been no data on using a new condom between oral, vaginal and anal sex among Australian FSWs; and therefore, further studies are required to explore the patterns of condom use in this population.

A substantial proportion of FSWs performed fellatio on men after condomless vaginal and anal sex which carries a significant risk of genital-to-oropharyngeal STI transmission (15). While we did not investigate condom use during oral sex in our study, previous studies suggest that one-fifth of FSWs undergo condomless fellatio with their male clients in the average working week (11), and FSWs are more likely to have condomless sex with their non-paying regular partners (16). Multi-site infection of gonorrhea and chlamydia is common in MSM and is also common in heterosexuals and FSWs (17–19). Chow et al. reported that the prevalence of oropharyngeal gonorrhea (2%) was more common than genital gonorrhea (1%) among FSWs in Melbourne in 2017, and the cause of this finding is unclear (17). While past research has shown that condomless fellatio is not a risk factor for oropharyngeal gonorrhea amongst FSWs, emerging evidence has suggested that tongue kissing may be a risk factor for oropharyngeal gonorrhea amongst the MSM population (20, 21). As almost all FSWs engaged in tongue-kissing during their most recent group sex event, this has potential to be a large cause of oropharyngeal gonorrhea transmission among FSWs and their clients. As there have been no studies examining the association between tongue-kissing and oropharyngeal STIs among FSWs in Australia, further studies may be needed to investigate the significance of this among this population, specifically tongue-kissing on oropharyngeal gonorrhea infection.

Our study has several limitations. First, this study was conducted at a single urban sexual health clinic, sex workers attending the sexual health clinic are more likely to work at licensed brothels and have regular 3-monthly HIV/STI testing as per the Sex Work Law. Sex workers who do not attend sexual health clinics for regular HIV/STI testing or work outside the legal framework may have different sexual practices. Additionally, our response rate was low (22%) and we do not know if their most recent group sex event was work-related. Thus, our findings may not be generalizable to all FSWs in Melbourne and settings where sex work is not legal or regulated. Furthermore, as our study period was limited by the COVID-19 pandemic, and our sample only contained only 68 FSWs who engaged in group sex, this study may be limited by its smaller sample size. Second, social desirability bias might have occurred when FSWs reported condom use or other activities including group sex, which may have underestimated the proportion of group sex or overestimated the proportion of condom use. We asked FSWs whether their male partners had changed condoms between partners, and participants may inaccurately report condom use, especially in a large group sex size when many people are involved due to recall bias. Furthermore, we also did not ask whether the men changed condoms between vaginal and anal sex. Third, we only asked for condom use during vaginal sex and anal sex but did not ask about condom use for fellatio or dental dam for cunnilingus. While condom use during fellatio among FSWs is reportedly lower than vaginal and anal sex, it is unknown if a great proportion of FSWs used condoms (or dental dam for cunnilingus) during oral sex if they did not use condoms for anal or vaginal sex (11). Further studies may be warranted to investigate this relationship further, and investigate the role of condom use has on prevention of oropharyngeal STI acquisition. Fourth, other sexual activities such as rimming are also commonly reported among FSWs and are associated with STIs but we did not collect this information (22).

Our study found over a quarter (27.5%) of FSWs had engaged in group sex in the past 3 months. Although most FSWs reported that they use condoms with their male partners during vaginal and anal sex in the most recent group sex event, about 16–20% of their male partners did not change condoms between every woman which put the women at risk of STIs by using the same condom.

Current safe sex messages focus on condom use during sex, using a new condom between oral, vaginal and anal sex, as well as between partners if there are more than two participants in the sexual activity, and our study highlights that such messages should be further reinforced for HIV and STI prevention (12).

## Data Availability Statement

The datasets presented in this article are not readily available because data cannot be made publicly available in order to protect patient privacy as per the approved ethics requirement. Requests to access the datasets should be directed to EC, eric.chow@monash.edu.

## Ethics Statement

The studies involving human participants were reviewed and approved by Alfred Hospital Ethics Committee. Written informed consent for participation was not required for this study in accordance with the national legislation and the institutional requirements.

## Author Contributions

EC and CF conceived and designed the study. CL performed the data analyses and wrote the first draft of the manuscript. EC provided statistical advice and oversaw the project. KM assisted with the project management. CF, JT, TP, EA, KM, MC, CB, and EC assisted with data interpretation. All authors were involved in revising the manuscript for important intellectual content and approved the final version.

## Funding

CF and CB were supported by an Australian National Health and Medical Research Council (NHMRC) Leadership Investigator Grants (GNT1172900 and GNT1173361). EC was supported by an NHMRC Emerging Leadership Investigator Grant (GNT1172873). EA was supported by Australian Government Research Training Program (RTP) scholarship from Monash University and Research Entry Scholarship from the Chapter of Sexual Health Medicine, Royal Australasian College of Physician. JT was supported by Australian Government Research Training Program (RTP) Scholarship from Monash University.

## Conflict of Interest

The authors declare that the research was conducted in the absence of any commercial or financial relationships that could be construed as a potential conflict of interest.

## Publisher's Note

All claims expressed in this article are solely those of the authors and do not necessarily represent those of their affiliated organizations, or those of the publisher, the editors and the reviewers. Any product that may be evaluated in this article, or claim that may be made by its manufacturer, is not guaranteed or endorsed by the publisher.
